# Seismic Damage Identification Method for Curved Beam Bridges Based on Wavelet Packet Norm Entropy

**DOI:** 10.3390/s22010239

**Published:** 2021-12-29

**Authors:** Tongfa Deng, Jinwen Huang, Maosen Cao, Dayang Li, Mahmoud Bayat

**Affiliations:** 1 Jiangxi Province Key Laboratory of Environmental Geotechnical Engineering and Hazards Control, Jiangxi University of Science and Technology, Ganzhou 341000, China; Hjwshm@jxust.edu.cn (J.H.); cmszhy@hhu.edu.cn (M.C.); 2 School of Civil and Surveying & Mapping Engineering, Jiangxi University of Science and Technology, Ganzhou 341000, China; 3 Department of Engineering Mechanics, Hohai University, Nanjing 210098, China; dyl@hhu.edu.cn; 4 Department of Mechanical Engineering, University of South Carolina, Columbia, SC 29208, USA; mbayat@mailbox.sc.edu

**Keywords:** curved beam bridges, wavelet packet transform, damage identification, norm entropy, numerical simulation, seismic excitation

## Abstract

Curved beam bridges, whose line type is flexible and beautiful, are an indispensable bridge type in modern traffic engineering. Nevertheless, compared with linear bridges, curved beam bridges have more complex internal forces and deformation due to the curvature; therefore, this type of bridge is more likely to suffer damage in strong earthquakes. The occurrence of damage reduces the safety of bridges, and can even cause casualties and property loss. For this reason, it is of great significance to study the identification of seismic damage in curved beam bridges. However, there is currently little research on curved beam bridges. For this reason, this paper proposes a damage identification method based on wavelet packet norm entropy (WPNE) under seismic excitation. In this method, wavelet packet transform is adopted to highlight the damage singularity information, the $L_{p}$ norm entropy of wavelet coefficient is taken as a damage characteristic factor, and then the occurrence of damage is characterized by changes in the damage index. To verify the feasibility and effectiveness of this method, a finite element model of Curved Continuous Rigid-Frame Bridges (CCRFB) is established for the purposes of numerical simulation. The results show that the damage index based on WPNE can accurately identify the damage location and characterize the severity of damage; moreover, WPNE is more capable of performing damage location and providing early warning than the method based on wavelet packet energy. In addition, noise resistance analysis shows that WPNE is immune to noise interference to a certain extent. As long as a series of frequency bands with larger correlation coefficients are selected for WPNE calculation, independent noise reduction can be achieved.

## 1. Introduction

In interchange project and urban overpass traffic systems, due to the limitations of the terrain environment and the requirements of line aesthetics, curved beam bridges are frequently adopted for traffic interconnection in all directions, to ensure smooth road routes and to ease traffic congestion [1]. However, China is an earthquake-prone country with a wide distribution of earthquakes, because it faces the pacific rim seismic belt on the east and boarders with the Eurasian seismic belt on the south. Furthermore, there is a significant bending-torsion-shear coupling effect in curved beam bridges due to their bending characteristics [2], with a complex and changeable force state. Thus, curved beam bridges will inevitably be damaged to various extents when subjected to earthquakes, resulting in the decay of the resistance of the structural system. If no timely damage detection and remedial maintenance measures are implemented, the damage may accumulate as time goes by, eventually leading to serious bridge accidents. This not only hinders transportation and threatens the lives of drivers and passers-by, it also results in huge losses to the national economy [3]. Consequently, it is particularly important to effectively identify seismic damage to bridge structures.

Damage identification is an important part of structural health monitoring (SHM). It aims to detect the damage location by specific methods, to reasonably analyze the severity of damage, to estimate the health condition and the residual life of the structure, etc. [4]. Damage identification has been widely used in various fields. Damage detection methods based on the measured response data of advanced sensors have been a hot research topic in recent years [5]. The basic methods based on measured response can be divided into static damage identification and dynamic damage identification. Static damage identification [6,7,8,9], which uses static test data, is commonly applied in structural model experiments and field load tests. Nonetheless, the test conditions for static damage identification are more stringent, and temporary traffic closures are required; therefore, it is generally difficult to carry out on-the-spot tests at bridge sites. Dynamic damage identification [10,11,12,13] is a more widely used identification method in the field of modern civil engineering, especially dynamic damage identification based on modal changes. The principle of modal-based damage identification methods is to identify the location of damage through changes modal parameters (natural frequency [14,15], mode of vibration [16,17], curvature modal [18,19], strain modal [20,21], etc.), and these modal parameters can generally be obtained using the time domain decomposition technique (TDD) [22,23]. Nevertheless, in practical applications, it is difficult to tell whether the modal changes are caused by structural damage or by changes in environmental factors (e.g., noise, temperature, humidity) [4]. Damage identification based on time–frequency signal processing is also a method of dynamic damage identification [24], including time domain, frequency domain, and time–frequency domain. Digital signal processing is an important part of the engineering field, and the main signal processing methods include Fourier Transform, Windowed Fourier Transform, Short Time Fourier Transform, Wavelet Transform, and Hilbert–Huang Transform. Among them, Wavelet Transform (WT) is the most representative in SHM, and the literature includes the following representative studies: Abdulkareem et al. [25] used two-dimensional Continuous Wavelet Transform (CWT) to decompose the difference value of the first order mode shape of the steel plate before and after the damage, and to detect whether the steel plate was damaged according to the difference after decomposition. Xin et al. [26] pointed out that Improved Empirical Wavelet Transform (IEWT) can effectively identify the modal parameters of the structure in the operating state. They used an IEWT-based method to successfully identify the modal parameters of a seven-story steel frame structure. Zhu et al. [27] constructed a crack identification index based on WT to locate the opening crack position of Functionally Graded Materials (FGMs). Furthermore, Guo et al. [28] reported that the detail coefficients of WT were highly sensitive to damage. They applied these coefficients to successfully detect the location and severity of structural damage.

However, due to the short duration and large energy of seismic excitation, the excited dynamic response therefrom is supposed to be highly non-stationary and nonlinear. Damage information is often hidden in the high-frequency part of the response signal; nonetheless, WT only subtly decomposes the low-frequency part of the signal. To achieve continuous cascade decomposition in the high-frequency and low-frequency parts, an increasing number of researchers are developing dynamic damage identification methods based on wavelet packet transform (WPT). Zhang et al. [29] conducted research on the defect identification of prefabricated structures. They proposed three defect identification indexes based on WPT, and successfully identified the defects of prefabricated concrete frame structures under noise conditions. Zhao et al. [30] combined digital image correlation (DIC) and WPT, and perfectly realized the monitoring and early warning provision for micro-damage in reinforced concrete beams. Naderpour et al. [31] put forward a two-step algorithm to identify the modal parameters based on WPT. They verified the feasibility and validity of the algorithm on the basis of vibration experiments on a three-layer framework model. Rajendran et al. [32] indicated that WPT is an advanced time–frequency analysis tool that can effectively excavate and amplify the individual points in the signal. They precisely identified minor damage to the composite plate structure by WPT. Chen et al. [33] adopted WPT to construct the variation rate of variance (VRV) damage index. They carried out numerical simulations and laboratory experiments on a damaged beam structure, and the results showed that VRV could correctly locate crack damage in a simply supported beam in a noisy environment. Zhang et al. [34] used WPT to decompose the dynamic response; then they took the energy ratio of each node as the damage characteristic vector and employed a neural network to identify the location of structural damage. The experimental results indicated that WPT was able to effectively extract information on individual instances of damage. Based on WPT, Wang et al. [35] presented an Energy Curvature Difference (ECD) damage identification index. The results of the two numerical studies showed that the damage position could be determined by observing sudden peaks in the ECD curve. Additionally, a 5% degree of micro-damage could also be accurately identified. Law et al. [36] first processed the response signal excited by impact loading using WPT, then obtained the signal energy distribution according to wavelet packet energy, and finally detected the single and multiple damage scenarios of the reinforced concrete beam on the grounds of changes in energy distribution. Furthermore, Ren et al. [37] described a shear connection part looseness (damage) identification method based on WPT. On the basis of experiments, they revealed that the method was extremely sensitive to damage, and its applicability and feasibility for application at bridge sites was demonstrated.

In addition to the identification methods based on WPT, by combining WP and information entropy theory, some scholars have developed a wavelet entropy-based method for damage identification. Ren et al. [38] defined wavelet entropy, relative wavelet entropy, and wavelet time entropy, and numerical simulation and laboratory experiments showed that these three kinds of wavelet entropy could locate and identify damage; moreover, relative wavelet entropy did not require pre-damage response data. Diao et al. [39] constructed a kind of wavelet entropy to identify structural damage under seismic excitation. They verified the feasibility of wavelet entropy on the basis of model experiments using a numerical simulation of an offshore platform structure and a vibration table. Lee et al. [40] proposed a bridge structure damage identification method based on continuous relative wavelet entropy. They concluded that the continuous wavelet entropy algorithm had a reliable damage location ability. Li et al. [41] assessed building structure damage by establishing Wavelet Singular Spectrum Entropy (WSSE). An experiment carried out using a 1/3-scale wood structure model was performed, and verified the reliability of WSSE. In summary, wavelet entropy not only inherits the advantage of high resolution from WT, it also integrates the ability of information entropy to quantitatively characterize damage information. With increasing decomposition scale, wavelet entropy becomes more sensitive to damage, greatly improving the recognition accuracy and effectively avoiding noise interference. Based on these advantages, wavelet entropy is particularly suitable for bridge structure damage identification.

Existing damage detection methods mainly focus on linear bridges, and there are few studies addressing damage identification in more complex special-shaped bridges under seismic excitation. As a consequence, this paper takes Curved Continuous Rigid-Frame Bridges (CCRFBs) as the research target and puts forward a wavelet packet norm entropy (WPNE)-based method for the identification of damage to the bridge structure under seismic excitation. WPNE is a kind of wavelet entropy. The information for the full frequency band is highlighted by WPT, the damage information is extracted by the *L_p_* norm characteristic of wavelet coefficients, and the information characteristic is measured quantitatively by the information entropy. The damage index therefrom combines the advantages of WPT, *L_p_* norm, and information entropy, which greatly improves the sensitivity and accuracy of damage identification.

Li et al. [42] investigated the damage detection problem in Curved Continuous Girder Bridges (CCGBs) by using wavelet packet singular entropy (WPSE). The effectiveness of WPSE was validated on the basis of numerical experiments, indicating that it is applicable for the identification and localization of earthquake-induced damage in the piers of the CCGB under noisy conditions. Following the work of Li et al. [42], this study focuses on the SHM problem in another type of curved bridge, namely Curved Continuous Rigid-Frame Bridges (CCRFBs). CCRFBs feature a rigid connection between the deck and the piers that differs from that in the CCGB. Different from WPSE in nature, WPNE uses *L_p_* norm to extract damage information, endowing WPNE-based methods with new damage characterization capabilities. In addition, WPNE adds a valid *p* value selection process compared with WPSE, which strengthens the damage distinction ability of the index. In terms of anti-noise performance, the WPNE-based method has stronger noise immunity.

The rest of this paper is organized as follows: Section 2 introduces the basic theory of WPT, *L_p_* norm and information entropy. Section 3 constructs a damage index based on WPNE and introduces the identification steps of CCRFBs. Section 4 establishes a finite element model of the CCRFB and carries out the dynamic analysis of the CCRFB. The identification results of the CCRFB are in presented in Section 5. Section 6 analyzes the effect of seismic excitation and compares the identification performance based on the WPNE and tests the noise resistance of the damage index. The conclusions are presented in Section 7.

## 2. Basic Theory

### 2.1. Wavelet Packet Transform

WT can only break down the low-frequency part of the signal, and not the high-frequency part (detail part), so the damage information in the high-frequency part cannot be highlighted, while the WPT can achieve continuous cascade decomposition in the low-frequency part and the high-frequency part of the signal. Compared with WT, WPT has better time–frequency characteristics and local engraving capabilities, and therefore, WPT can focus on any detail bands of the signal, fully abstracting the valid information on the characteristics of the full frequency band. Any dynamic response signal x(t) after the j-layer WPT can be written as [43]:

(1) $$x\left(t\right)=\overset{2^{j}}{\underset{i=1}{\sum }}x_{j}^{i}$$

where $x_{j}^{i}$ is the dynamic response signal of each frequency band after decomposition and can be expressed as:

(2) $$x_{j}^{i}=\underset{k}{\sum }c_{j,k}^{i}{\cdot \psi }_{j,k,i}\left(t\right)$$

where $\psi_{j,k,i}\left(t\right)$ is a set of standard orthogonal wavelet basis functions. When r ≠ s,

(3) $$\psi_{j,k}^{r}\left(t\right){\cdot \psi }_{j,k}^{s}\left(t\right)=0$$

$c_{j,k}^{i}$ is defined as the wavelet packet coefficient at the *j* decomposition scale and can be expressed as:

(4) $$c_{j,k}^{i}=\int_{-\infty }^{+\infty }x\left(t\right){\cdot \psi }_{j,k,i}\left(t\right)dt$$

The wavelet packet coefficient matrix, composed of the coefficient $c_{j,k}^{i}$, reflects the information of the response signal in each band. In 2*^j^* bands, some specific bands (especially the high-frequency bands) conceal information on individual instances of damage. 

### 2.2. L_p_ Norm

In mathematics, the norm is a function of the concept of length in vector space, and meets three conditions: positive definiteness, homogeneity, and subadditivity. The most commonly used norm in normed linear space is the *L_p_* norm. If ${X=[x}_{1}{,x}_{2}{,x}_{3}{,\ldots ,x}_{n}{]}^{T}$, the *L_p_* norm of vector X can then be written as [44]:

(5) $${\left\|X\right\|}_{p}={\left(\overset{n}{\underset{i=1}{\sum }}{\left|x_{i}\right|}^{p}\right)}^{\frac{1}{p}}$$

Theoretically *p* value is [0,+∞], but when 0 ≤ p <1, it does not meet the subadditivity condition, so strictly it is not *L_p_* norm. Values of 1, 2 or +∞ are often taken as the *p* value. When *p* = 1, the norm *L*_1_ is also known as the Manhattan Distance, which is capable of characterizing the difference between different vectors and clearing the features without information and meaning, thereby achieving the sparsity of the vector. When *p* = 2, the norm *L*_2_ is also known as the Euclidean Distance, which can also express the difference between different vectors, but *L*_2_ is generally used to optimize the regularization item of the target function. The $L_{\infty }$ norm is able to extract the largest element in the vector.

### 2.3. Information Entropy

Information entropy is the average information integration after redundancy is removed from the information, and is capable of quantifying the abstract concept of information and characterizing the degree of uncertainty of events. If it is assumed that there are n source signals with values, and the probabilities of their occurrence are $p_{1}{,\;p}_{2}{,\ldots p}_{n}$, the degree of uncertainty of the source signal can be expressed as:

(6) $$H=\overset{n}{\underset{i=1}{\sum }}-p_{i}{log}_{2}\left(p_{i}\right)$$

Information entropy represents the expectation of the total amount of information in a system. The more complex the system is, the higher the degree of uncertainty will be and the greater the information entropy will be. Conversely, the simpler the system is, the lower the degree of uncertainty will be and the smaller the information entropy will be. In the field of SHM, many scholars have applied the theory of information entropy when researching structural damage identification [45].

## 3. Damage Identification Method

### 3.1. Damage Identification Index

The dynamic response signal x(t) measured by the sensor is decomposed by WPT with a decomposition scale of *j*. After decomposition, $2^{j}$ frequency bands and wavelet packet coefficients of each band $\left\{c_{j}^{1}\left(t\right){,\;c}_{j}^{2}\left(t\right){,\;\ldots ,\;c}_{j}^{n}\left(t\right)\right\}$ are obtained, where ${n=2}^{j}$, and ${\;c}_{j}^{i}\left(t\right)$ is a multidimensional vector, which indicates wavelet package coefficients of node in layer *j* of wavelet tree, and can be written as:

(7) $$c_{j}^{i}\left(t\right)={\left[c_{j,1}^{i}{,\;c}_{j,2}^{i}{,\;\ldots ,\;c}_{j,m}^{i}\right]}^{T}$$

For *L_p_* norm of $c_{j}^{i}\left(t\right)$, its expression is:

(8) $$\|c_{j}^{i}{\left(t\right)\|}_{p}={\left(\overset{m}{\underset{l=1}{\sum }}{\left|c_{\mathrm{jl}}^{i}\right|}^{p}\right)}^{\frac{1}{p}}$$

For convenience of expression, $\|c_{j}^{i}{\left(t\right)\|}_{p}$ is denoted as $L_{p}^{i}$. By combining $L_{p}^{i}$ with the theory of information entropy, the damage characteristic factor WPNE can be constructed, with a specific expression as follows:

(9) $$WPNE=\overset{2^{j}}{\underset{i=1}{\sum }}-\lambda_{i}{log}_{2}\left(\lambda_{i}\right)$$

where

(10) $$\lambda_{i}=\frac{L_{p}^{i}}{\sum_{i=1}^{2^{j}}L_{p}^{i}}$$

WPNE integrates the advantages of WPT, *L_p_* norm and information entropy, which are embodied in: (1) the high-resolution characteristics of the WPT are used to achieve more detailed decomposition of nonlinear and non-stable response signals, highlighting the information of the full band; (2) *L_p_* norm is applied to the abstract effective damage characteristics and the sparse useless interference feature; (3) the degree of uncertainty of the information system is quantitatively characterized by information entropy. In summary, WPNE is capable of highlighting detail, extracting features and quantifying information. Based on WPNE, we can construct the structural damage identification index ${DI}_{WPNE}$; the index is defined as follows:

(11) $${DI}_{WPNE}=\left|\frac{{WPNE}^{h}{\;-\;WPNE}^{d}}{{WPNE}^{h}}\right|$$

where *WPNE^h^* and *WPNE^d^* are WPNE in the state of structural health and damage, respectively. According to Formula (11), *DI_WPNE_* reflects the relative amount of the change before and after the damage, moreover the size of the change represents different states of the structure, that is, health or damage state. When the structure is not damaged or is slightly damaged, *DI_WPNE_* is zero or close to zero and the *DI_WPNE_* curve is relatively flat. When the structure is damaged to a certain extent, the value of *DI_WPNE_* at the damage position is a positive number greater than zero, and the *DI_WPNE_* curve shows a significant mutation at the damage position, displaying a sudden peak. The greater the severity of the damage, the greater the value of the peak. Therefore, the *DI_WPNE_* index is capable of identifying the location of the damage and characterizing the severity of the damage.

To further judge the damage location reasonably and accurately and provide a damage warning, the damage threshold ${\mathrm{DI}}_{\mathrm{WPNE}}^{\mathrm{TH}}$ is introduced on the basis of the principle of the unilateral confidence interval [38], and the damage warning index *EW_WPNE_* is established on the basis of the difference between the *DI_WPNE_* index and the damage threshold ${\mathrm{DI}}_{\mathrm{WPNE}}^{\mathrm{TH}}$:

(12) $${\mathrm{EW}}_{\mathrm{WPNE}}{\;=\mathrm{DI}}_{\mathrm{WPNE}}{\;-\mathrm{DI}}_{\mathrm{WPNE}}^{\mathrm{TH}}$$

where

(13) $${\mathrm{DI}}_{\mathrm{WPNE}}^{\mathrm{TH}}{\;=\mu +u}_{\alpha }\left(\frac{\sigma }{\sqrt{n}}\right)$$

where *n* is the total number of measurement points, and μ and σ are the average and standard deviation of the *DI_WPNE_* index value of all measurement points, respectively; $u_{\alpha }$ is the upper α quantile of the standard normal distribution, α is commonly referred to as the significance level, which generally takes on 0.05, 0.02, 0.015 or other small values, (1−α) is called confidence probability, where α has a value of 0.02 in this paper, and the corresponding confidence probability is 98%. By checking the upper α quantile table of the standard normal distribution, $u_{0.02\;}=2.06$ can be found. 

The advantages of setting the damage threshold are as follows: (1) when the *DI_WPNE_* index value of the measurement point is greater than the damage threshold ${\mathrm{DI}}_{\mathrm{WPNE}}^{\mathrm{TH}}$, *EW_WPNE_* > 0, the health monitoring system will 98% believe that damage will appear at the measurement point, and the system will provide an early warning; (2) when the *DI_WPNE_* index value of the measurement point is not greater than the damage threshold ${\mathrm{DI}}_{\mathrm{WPNE}}^{\mathrm{TH}}$, ${\mathrm{EW}}_{\mathrm{WPNE}}\;\leq \;0$, the health monitoring system will 98% believe that the structure is not damaged, and the system will not provide a warning. Theoretically, a zero value of *EW_WPNE_* can be used as the damage warning value. However, in the course of practical application, in order to reduce false reports caused by environmental factors (e.g., temperature, noise, and humidity), initial micro-damage, and measurement errors, numbers greater than zero are usually taken as the damage warning value.

### 3.2. Damage Identification Steps

This paper primarily investigates seismic damage identification for CCRFBs, and the main identification steps are as follows:

Step 1. Select the appropriate measurement point location and lay sensors according to the structural form of CCRFBs and test needs.

Step 2. Determine the seismic damage location and set reasonable damage scenarios based on the results of the vulnerability analysis.

Step 3. Apply ground motion acceleration in the direction of the most unfavorable seismic input of curved beam bridges and measure the dynamic response of each measurement point before and after damage.

Step 4. Take the energy entropy as the cost function to determine the optimal wavelet packet parameters (wavelet basis function and decomposition scale).

Step 5. Compare the damage identification effect of different dynamic responses and select the best dynamic response.

Step 6. Select the *p* value of WPNE according to the damage identification accuracy and calculation efficiency.

Step 7. Decompose the response signal before and after structural damage with WPT, calculate the damage characteristic factor WPNE to obtain *DI_WPNE_* index, introduce the damage threshold ${\mathrm{DI}}_{\mathrm{WPNE}}^{\mathrm{TH}}$ to further obtain the damage warning index *EW_WPNE_*, determine the damage position according to the sudden peak of the index curve, and identify the severity of damage using the peak value.

Step 8. Compare with *D_er_* (wavelet packet energy ratio change rate index). Add white Gaussian Noise with different signal-to-noise ratios to analyze the noise resistance of the damage identification index.

**Figure 1:**
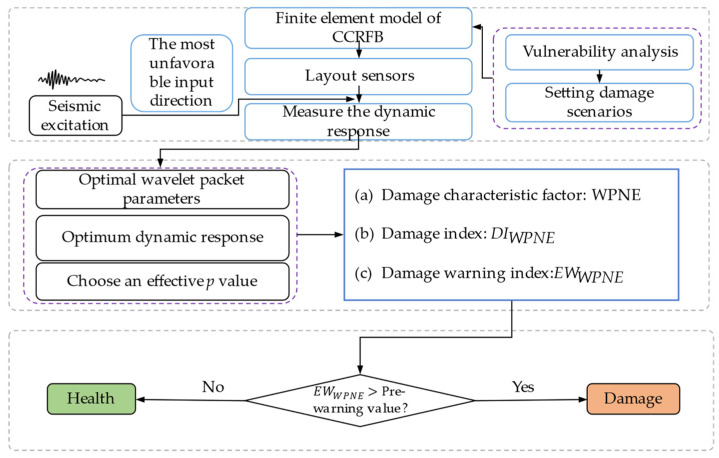
Flowchart of seismic damage identification for CCRFBs.

The specific identification flowchart is shown in Figure 1.

## 4. Dynamic Analysis of CCRFB

### 4.1. Establish a CCRFB Finite Element Model

**Figure 2:**
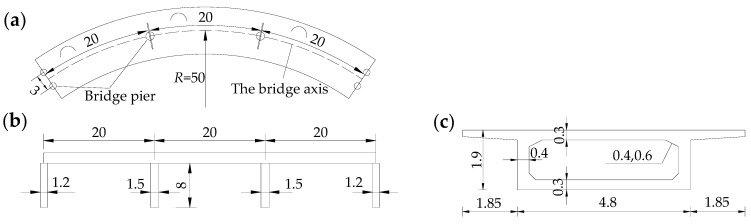
Geometric dimensions of the CCRFB: (**a**) plane graph; (**b**) elevation graph; (**c**) main girder cross section.

**Figure 3:**
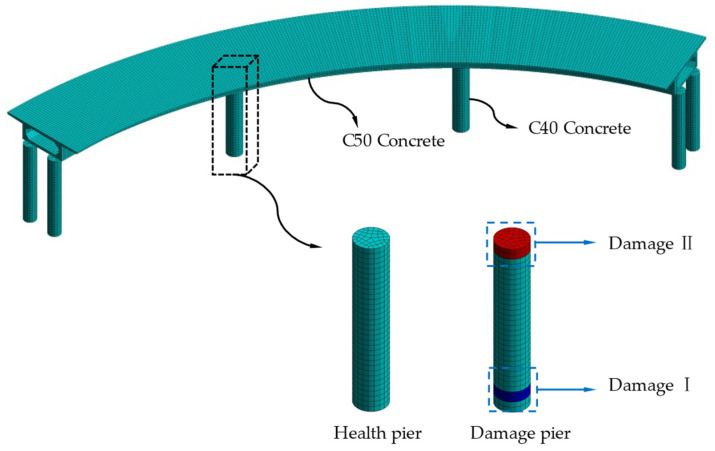
Finite element model of the CCRFB and damage locations.

The geometric dimensions of the CCRFB are shown in Figure 2. It has a structure with a three-span single-box beam, with two bridge piers with a radius of 1.2 m at the edge span and one pier with a radius of 1.5 m in the span; the finite element model of the CCRFB (see Figure 3) was established using the large-scale universal finite element software ANSYS, and the model was discretized into 34,501 SOLID elements. The material parameters were as follows: the upper structure uses C50 concrete (material density of ${2500\;\mathrm{kg}/m}^{3}$, elastic modulus of $3{.45\;\times \;10}^{4}\;\mathrm{MPa}$, and Poisson’s ratio of 0.2). The lower structure of the CCRFB uses C40 concrete (material density of ${2500\;\mathrm{kg}/m}^{3}$, elastic modulus of $3{.25\;\times \;10}^{4}\;\mathrm{MPa}$, and Poisson’s ratio of 0.2). The boundary conditions were as follows: the rotational and translational degrees of freedom of all nodes are constrained at the bottom of the pier.

### 4.2. Set Damage Scenarios

On the basis of a large number of engineering examples and seismic vulnerability analyses, it can be observed that the position of seismic damage in curved beam bridges is generally located at the bottom of the bridge pier (lower damage; I) and the pier–beam connection (upper damage; II). Furthermore, damage I and II do not appear at the same time. If one appears, the other one will not appear, that is, the two are characterized by mutual exclusivity. Therefore, in this paper, only a single instance of damage is considered, and damage is set at bridge pier No. 3 (see Figure 3). Damage is simulated by reducing the element stiffness (reducing Elastic Modulus) [46], with a damage severity of 5%–35% at each damage location, including non-damaged scenario 1, for a total of 15 sets of scenarios, as detailed in Table 1.

### 4.3. Enter Ground Motion Acceleration 

**Figure 4:**
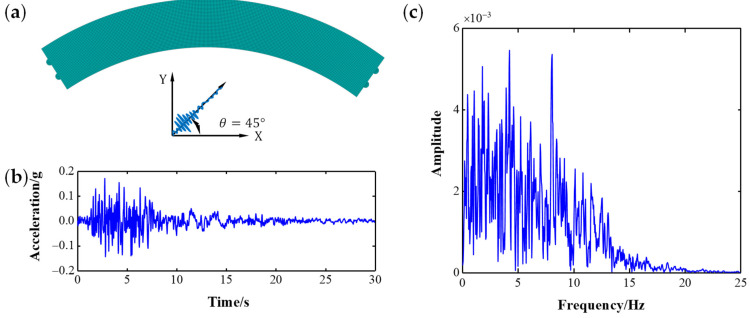
Input seismic excitation: (**a**) the most unfavorable input direction; (**b**) SF accelerogram; (**c**) Fourier spectrum of SF.

The entered angle of seismic excitation significantly affects the maximum dynamic response of curved beam bridges. For this reason, the most unfavorable input angle of seismic excitation for CCRFBs is θ=45°, in accordance with engineering examples and our own experience. Therefore, the San Fernando (simplified SF) input ground motion acceleration was entered in the direction of 45° by applying inertial force. The entered direction and the time–frequency domain of SF are shown in Figure 4. 

### 4.4. Measured Dynamic Response

**Figure 5:**
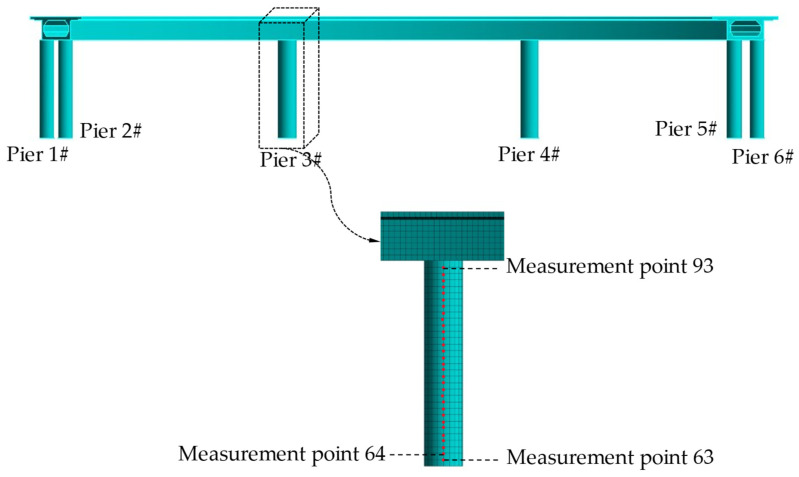
Layout of measurement points.

**Figure 6:**
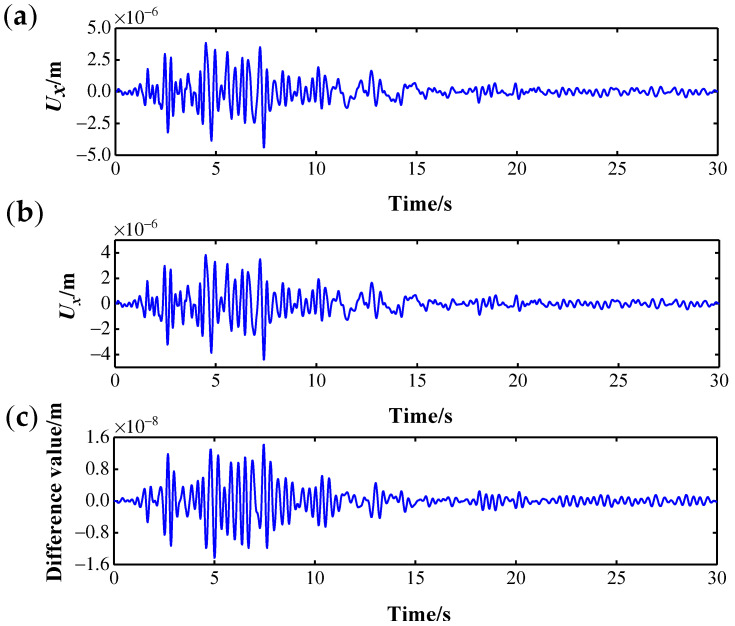
Displacement response of No. 64 measurement point: (**a**) scenario 1; (**b**) scenario 2; (**c**) the response difference.

For each bridge pier, 31 measurement points are arranged from bottom to top, and displacement sensors in the *x* and *y* directions are installed to measure the displacement dynamic response data of the corresponding measurement points. See Figure 5 for the measurement point layout, where the serial numbers of the measurement point of bridge pier No. *n* range from 1+31(n−1) to 31n. Figure 5 only shows the serial numbers of the measurement points on the 3rd pier. In accordance with Section 4.2, damage is set on bridge pier No. 3 only, so the serial numbers of the measurement points for damage I are (64, 65, 66) and the serial numbers of measurement points for damage II are (92, 93). Figure 6 shows the $U_{x}\;(x$-directional displacement) response and difference of No. 64 measurement point in scenario 1 and scenario 2, and it is impossible to judge whether there is damage by directly observing the difference in the response signal.

## 5. Damage Identification for CCRFB

### 5.1. Choose Optimal Dynamic Response

**Figure 7:**
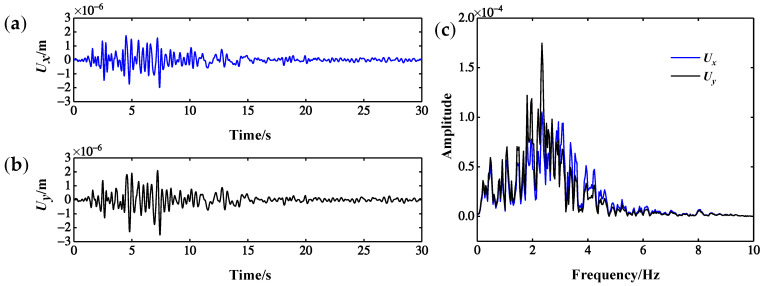
Displacement response of No. 63 measurement point under scenario 1: (**a**) *U_x_*; (**b**) *U_y_*; (**c**) Fourier spectrum.

**Figure 8:**
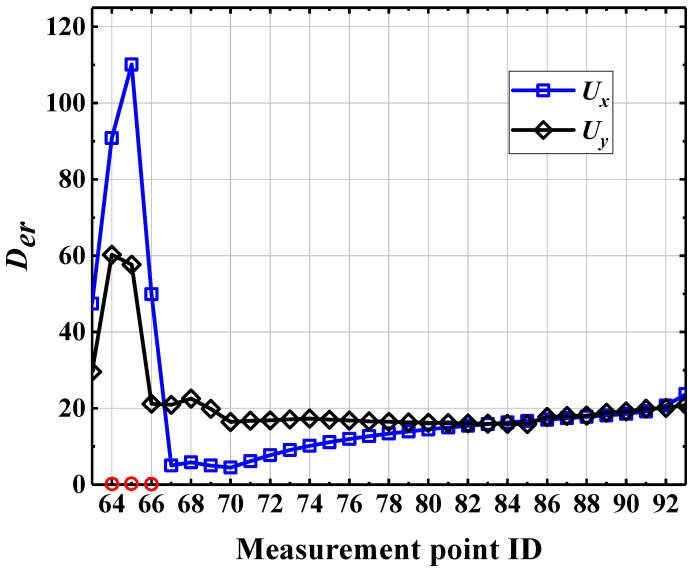
Comparison of *U_x_* and *U_y_* response identification results.

There are many kinds of dynamic responses, such as displacement, velocity and acceleration response. Theoretically, vibration-based damage identification methods generally universal for different dynamic responses, but different dynamic responses often vary with respect to the accuracy and sensitivity of damage identification. Therefore, selecting a damage-sensitive dynamic response can further improve the damage identification ability of the index. In Section 4.4, *U_x_* and *U_y_* response data for each point of the CCRFB were extracted. The No. 63 measurement point of scenario 1 is taken as an example; Figure 7 presents the *U_x_* and *U_y_* response of the measurement point and the corresponding frequency spectrum. Obviously there is no significant difference between *U_x_* and *U_y_* response in the time frequency domain, so it is hard to judge the sensitivity of *U_x_* and *U_y_* response to damage. To select a displacement response that is more sensitive to damage, according to the existing damage identification index *D_er_* (wavelet package energy ratio change rate), the damage identification effects of *U_x_* and *U_y_* response under scenario 2 are compared. For a definition of *D_er_*, please see Formula (14) [47]. For the results of *D_er_* index damage identification based on *U_x_* and *U_y_* response, please see Figure 8.

(14) $$D_{\mathrm{er}}=\sum_{i=1}^{2^{j}}\frac{\left|I_{i}^{h}-I_{i}^{d}\right|}{I_{i}^{h}}$$

where *j* is the decomposition level; $I_{i}^{h}$ and $I_{i}^{d}$ are ratios between sub-band energy and energy mean before and after damage, respectively.

The serial numbers of the measurement point of the damage elements under scenario 2 are (64, 65, 66). The following can be found from Figure 8: (1) the *D_er_* index value of the damage identification based on *U_x_* and *U_y_* response is larger at the damage location, and the *D_er_* curve shows a significant mutation in the damage area with an obvious peak, but it is relatively flat and smooth in other non-damaged positions. (2) The *D_er_* index value of damage identification based on *U_y_* response at the No. 66 measurement point of the damage is not much different from that of the *D_er_* index value at the non-damaged measurement point. (3) Compared with the $U_{y}$ response, damage identification based on the *U_x_* response is more prominent at the damage position. On the whole, the damage identification effect based on the *U_x_* response is better. Therefore, the *U_x_* response is selected below for structural damage identification research.

### 5.2. Select Optimal Wavelet Packet Parameters

Selecting the optimal wavelet packet parameters is a prerequisite for ensuring accurate and reliable identification results. Before WPT, it is necessary to determine the wavelet packet parameters, that is, the wavelet basis function and the decomposition scale. The accuracy of the identification method will be reduced if the parameters are not selected properly. At present, there is no unified theoretical method for the selection of the optimal wavelet basis functions. In the field of damage monitoring, the most commonly used selection method is to select a set of base functions to be determined first based on the property of the base function (vanishing moment, support length, orthogonality) and signal characteristics, then to construct a cost function *M* with energy entropy, next to calculate the *M* value of the base function to be determined under the same signal, and finally to select the basis function [48] with a relatively small *M* as the optimal wavelet base function. For the decomposition scale, the larger the decomposition scale of the same wavelet packet base function, the finer the frequency band division after decomposition, which can improve the calculation accuracy, to a certain extent. However, an excessive decomposition scale will lead to a great deal of information redundancy, as well as requiring a long calculation time. Therefore, it is necessary to comprehensively consider the calculation results and calculation efficiency in order to select the optimal decomposition scale. The cost function *M* is defined as follows [49]:

(15) $$M=\overset{2^{j}}{\underset{k=1}{\sum }}-P_{k}{\mathrm{lg}(P}_{k})$$

where *j* is the decomposition scale and $P_{k}$ is the energy ratio of frequency band No. *k* after normalization, that is, $P_{k}{=E}_{j}^{k}\left(t\right)/\overset{2^{j}}{\underset{k=1}{\sum }}E_{j}^{k}\left(t\right)$; after WPT, the $2^{j}$ frequency bands can be obtained, and $E_{j}^{k}\left(t\right)$ is the wavelet packet energy of frequency band No. *k*.

**Figure 9:**
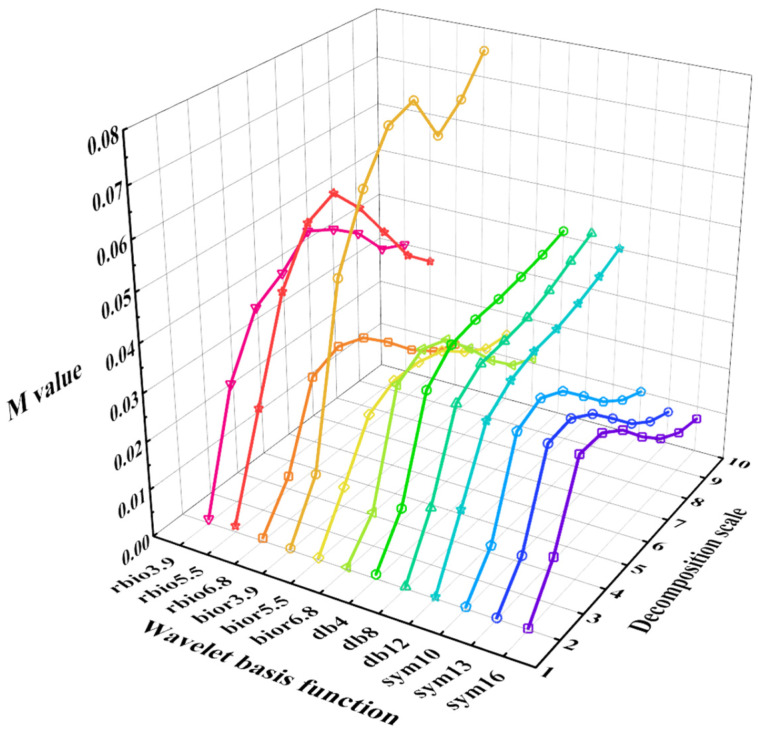
The cost function *M* value of the basis function to be determined.

dbN, bior $N_{r}.N_{d}$, rbio $N_{r}.N_{d}$ and symN in the wavelet family, which have a unique ability to extract features, can be used as the base function in the field of damage detection. Therefore, in this paper, rbio3.9, rbio5.5, rbio6.8, bior3.9, bior5.5, bior6.8, db4, db8, db12, sym10, sym13, and sym16 are selected as the wavelet basis function to be determined. To select the optimal wavelet basis function, according to Formula (15), the *U_x_* response extracted from the No. 63 measurement point under scenario 1 under SF seismic excitation is used to calculated the *M* value of the base function to be determined, with a decomposition scale of 2–10. The results are shown in Figure 9, on the basis of which we can see that the *M* value of sym13 in all the wavelet packet basis functions to be determined is relatively small overall, so it is necessary to choose the sym13 wavelet as the optimal wavelet basis function, compare the identification accuracy of the sym13 wavelet at different scales, and consider the calculation efficiency; finally, an optimal decomposition scale of 7 was chosen. 

### 5.3. Select Valid p Values

**Figure 10:**
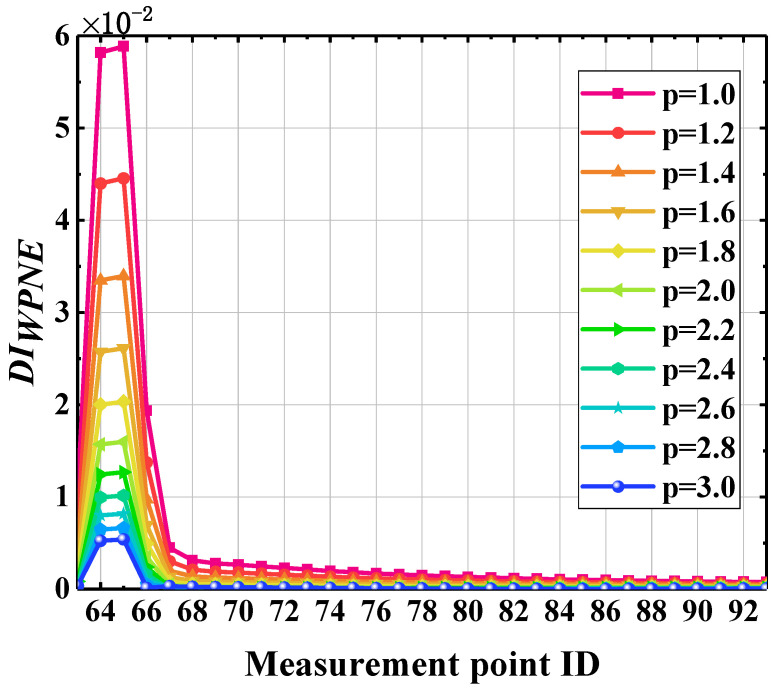
Identification results of different *p* values.

The *L_p_* norm is introduced in the construction of WPNE. According to Formula (8), the damage identification indexes based on WPNE can be further determined only by selecting the appropriate *p* value, but there is no uniform standard for the determination of *p* values. When *p* is 2, the *L*_2_ norm of the band is essentially the square root of the wavelet package energy. Theoretically, indexes of *p* greater than or equal to 1 are valid, but the larger the *p* value is, the longer the corresponding calculation time will be. Therefore, under the premise of ensuring the accuracy of damage identification, smaller values should be taken for *p* to improve calculation efficiency. To select the most effective *p* values, it is necessary to compare the damage identification effect under the conditions of scenario 2 when p=0.2 × n+0.8 (n=1,2,3,…,11). A comparison of the damage identification results is provided in Figure 10, on the basis of which it can easily be found that when p ≥ 1, *DI_WPNE_* shows a mutation in the damage position, with a peak; *DI_WPNE_* is always able to accurately identify the location of the damage. Nevertheless, the larger the *p* value, the smaller the peak value will be, and the longer the calculation time will be. Hence, it is necessary to comprehensively consider calculation efficiency, damage identification sensitivity, and damage positioning accuracy, and thus 1 is selected as a valid *p* value. The following analysis is based on WPNE when *p* = 1.

### 5.4. Damage Identification Results

**Figure 11:**
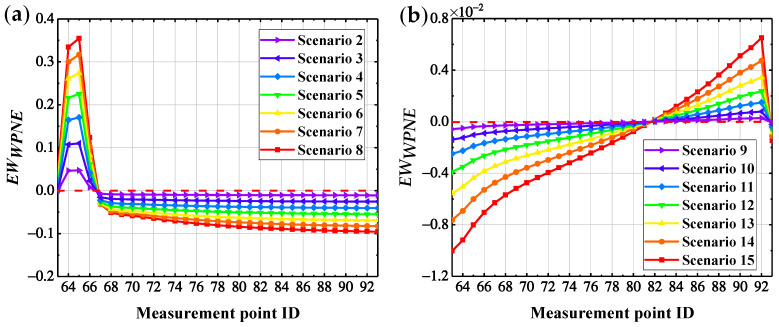
*EW_WPNE_* damage identification results: (**a**) damage I; (**b**) damage II.

In accordance with Section 5.2, the sym13 wavelet basis function is selected, with a decomposition scale of 7, the *U_x_* response measured at the No. 63–93 measurement points on bridge pier No. 3 of the CCRFB before and after the occurrence of damage is decomposed by the wavelet packet, then *DI_WPNE_* is calculated when *p* is equal to 1, and the damage early warning index *EW_WPNE_* is further calculated in combination with the damage threshold ${\mathrm{DI}}_{\mathrm{WPNE}}^{\mathrm{TH}}$. Figure 11 shows the identification results of the CCRFB under SF seismic excitation. In the picture, scenarios 2–8 incorporate lower damage I, and scenarios 9–15 incorporate upper damage II.

According to Section 3.1, the area where ${\mathrm{EW}}_{\mathrm{WPNE}}\;>0$ is the damage position, and the area where ${\mathrm{EW}}_{\mathrm{WPNE}}\;\leq \;0$ is the non-damaged position. According to Figure 11, for lower damage I, *EW_WPNE_* is greater than zero at the damage position, with a peak, and is lower than zero at non-damaged locations. Furthermore, *EW_WPNE_* increases as the severity of damage increases; therefore, *EW_WPNE_* can accurately locate the lower damage I and characterize the severity of the damage. For upper damage II, although *EW_WPNE_* is greater than zero at the location of the damage, with a peak, *EW_WPNE_* is also greater than zero in some non-damaged positions close to the damage. Accordingly, *EW_WPNE_* is not suitable for identifying upper damage II.

**Figure 12:**
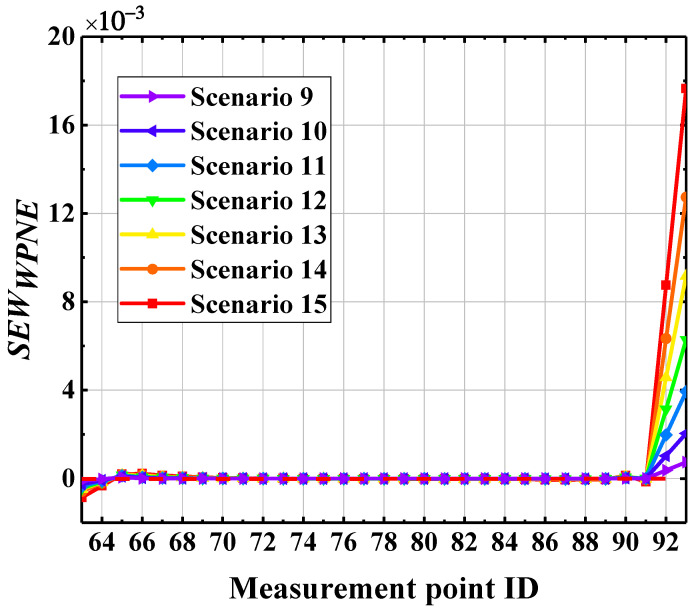
Modified identification results of damage II using index *SEW_WPNE_*.

To further improve the ability of the *EW_WPNE_* index to identify upper damage II, it is necessary to take into account the peak at the upper damage measurement point and good curve continuity in non-damaged areas. According to the central difference principle, the upper damage correction index *SEW_WPNE_* is constructed to magnify the curve mutation and remove the holistic trend of the curve, further highlighting the damage position. The definition of *SEW_WPNE_* is shown in Formula (16), below, and the damage identification results of upper damage II using the correction index *SEW_WPNE_* are shown in Figure 12.

(16) $${\mathrm{SEW}}_{\mathrm{WPNE}}\left(i\right)=-\frac{{\mathrm{EW}}_{\mathrm{WPNE}}\left(i-1\right)-{2\mathrm{EW}}_{\mathrm{WPNE}}\left(i\right){+\mathrm{EW}}_{\mathrm{WPNE}}\left(i+1\right)}{{\left(\Delta x\right)}^{2}}$$

where *i* is the serial number of the measurement points, Δx is the difference of the serial number of the adjacent measurement points, and here Δx=1. *SEW_WPNE_* essentially refers to the second-order numerical differentiation of *EW_WPNE_*, through which the singular point of *EW_WPNE_* is prominent in the form of numerical differentiation. Formula (16) can only calculate the *SEW_WPNE_* value of non-endpoints. For the No. 63 and No. 93 measurement points at the endpoint, Formulas (17) and (18) are used to calculate the *SEW_WPNE_* value.

(17) $${\mathrm{SEW}}_{\mathrm{WPNE}}\left(63\right)=-\frac{{2\mathrm{EW}}_{\mathrm{WPNE}}\left(63\right)-{5\mathrm{EW}}_{\mathrm{WPNE}}\left(64\right){+4\mathrm{EW}}_{\mathrm{WPNE}}\left(65\right)-{\mathrm{EW}}_{\mathrm{WPNE}}\left(66\right)}{{\left(\Delta x\right)}^{2}}$$

(18) $${\mathrm{SEW}}_{\mathrm{WPNE}}\left(93\right)=-\frac{{2\mathrm{EW}}_{\mathrm{WPNE}}\left(93\right)-{5\mathrm{EW}}_{\mathrm{WPNE}}\left(92\right){+4\mathrm{EW}}_{\mathrm{WPNE}}\left(91\right)-{\mathrm{EW}}_{\mathrm{WPNE}}\left(90\right)}{{\left(\Delta x\right)}^{2}}$$

It can be concluded from Figure 12 that the correction index *SEW_WPNE_* is only greater than 0 at damage measurement points (30, 31), and increases with increasing damage severity, while ${\mathrm{SEW}}_{\mathrm{WPNE}}\;\leq \;0$ at other non-damaged measurement points. Therefore, for upper damage II, the *SEW_WPNE_* index has a strong capacity for damage identification and positioning, as well as the ability to characterize the severity of structural damage. Unfortunately, due to the introduction of the second-order central difference algorithm to the calculation of *SEW_WPNE_*, computational noise may be introduced, thereby reducing the immunity of the *SEW_WPNE_* index to noise interference. The noise resistance of the *EW_WPNE_* and *SEW_WPNE_* indexes will be discussed in detail in the next section.

From the practical application of the dynamic damage detection method, it can be found that the initial minor damage, environmental noise and measurement errors of the structure will have a certain impact on the identification process. Therefore, the zero value in the ideal state will not be taken as the damage warning value. Instead, values greater than zero are commonly taken. To identify minor damage below 5%, 80% of *EW_WPNE_* and *SEW_WPNE_* index values at the 5% damage severity are taken as the damage warning value for damage I and II, respectively. The damage warning values for the CCRFB are shown in Table 2. 

**Figure 13:**
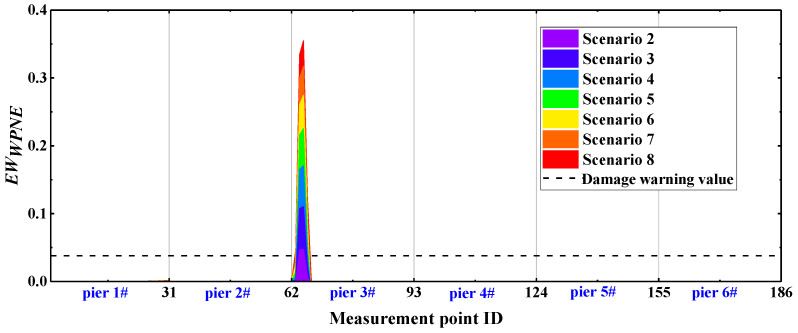
*EW_WPNE_* identification results of damage I of 6 bridge piers.

**Figure 14:**
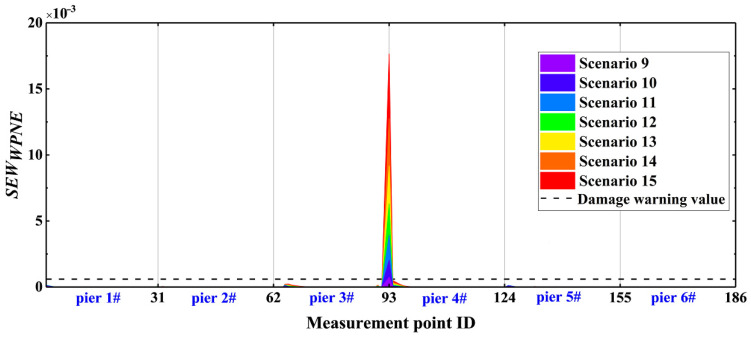
*SEW_WPNE_* identification results of damage II of 6 bridge piers.

On the basis for a comparison of Figure 11 and Figure 12, the *EW_WPNE_* index value of the lower damage and *SEW_WPNE_* index value of the upper damage are not on the same order of magnitude. Furthermore, Table 2 shows that the value of the lower damage warning is about 60 times that of the upper damage warning value, so it is necessary to separate the upper damage identification from the lower damage identification and to consider them individually. The damage warning value of Table 2 is applied to the identification of all the CCRFB bridge piers (P1–P6). The damage I identification results are shown in Figure 13, and the damage II identification results are shown in Figure 14. The black dashed line in the figure refers to the damage warning value. According to Section 4.2, the damage position of the CCRFB is only set on the P3 pier, the serial numbers of the measurement points for damage I are (64, 65, 66), and the serial numbers of the measurement points for damage II are (92, 93). It is not difficult to see from the figure that both *EW_WPNE_* and *SEW_WPNE_* at the damage measurement point of the P3 pier exceed the damage warning value, and the damage index value increases with increasing damage severity. In other non-damaged piers, *EW_WPNE_* and *SEW_WPNE_* do not exceed the damage warning value, which is consistent with the actual situation. In summary, the WPNE-based damage identification index can accurately identify the damage position of the CCRFB and quantitatively characterize the severity of damage, thereby achieving structural damage detection, positioning and early warning.

## 6. Discussion

### 6.1. Compare Identification Index D_er_

**Figure 15:**
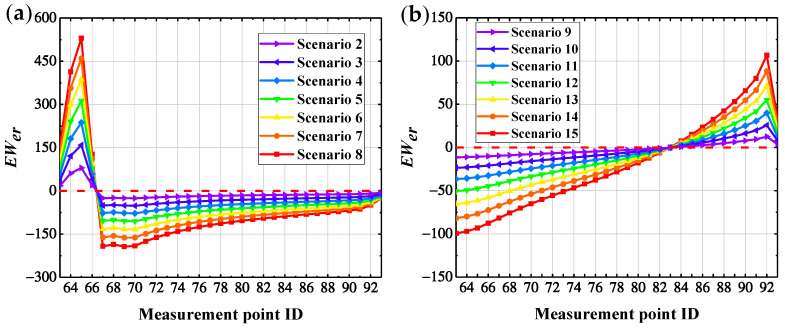
*EW_er_* damage identification results: (**a**) Damage I; (**b**) Damage II.

**Figure 16:**
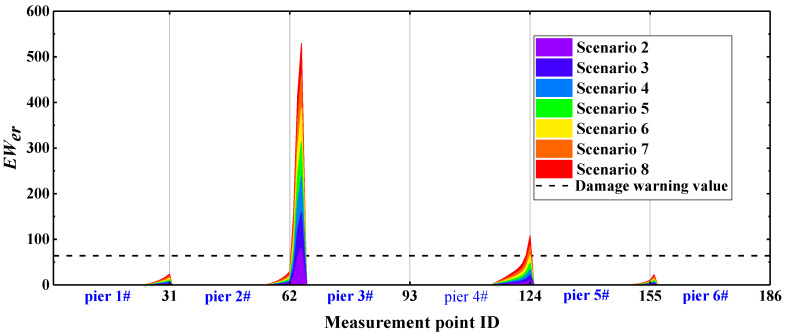
*EW_er_* identification results of damage I of 6 bridge piers.

**Figure 17:**
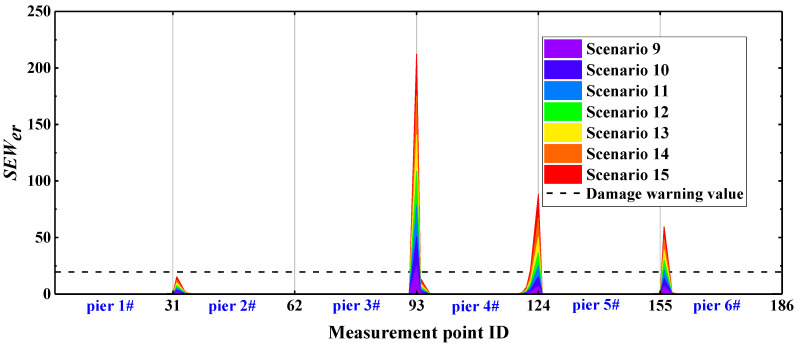
*SEW_er_* identification results of damage of 6 bridge II piers.

To compare and describe the identification performance of the damage index based on WPNE, it is necessary to compare it with the *D_er_* index. The definition of *D_er_* is detailed in Formula (14) in Section 5.1. *D_er_* identifies the damage position by means of the energy change of each band before and after the damage takes place. Similarly, according to Formulas (12) and (13), the damage threshold based on *D_er_* is set to further obtain the damage warning index ${\mathrm{EW}}_{\mathrm{er}}$. The identification results of ${\mathrm{EW}}_{\mathrm{er}}$ are shown in Figure 15. The identification results of ${\mathrm{EW}}_{\mathrm{er}}$ resemble those of *EW_WPNE_*. ${\mathrm{EW}}_{\mathrm{er}}$ can accurately identify lower damage I in the CCRFB. For upper damage II, the area where ${\mathrm{EW}}_{\mathrm{er}}\;>$ 0 is far beyond the damage measurement points, and ${\mathrm{EW}}_{\mathrm{er}}$ possesses a poor ability of detect damage II. Likewise, in accordance with the characteristics of the ${\mathrm{EW}}_{\mathrm{er}}$ curve changes, Formulas (16)–(18) were used to construct a correction index ${\mathrm{SEW}}_{\mathrm{er}}$ and introduce the damage warning value. ${\mathrm{EW}}_{\mathrm{er}}$ and ${\mathrm{SEW}}_{\mathrm{er}}$ were applied in the damage identification for all bridge piers in the CCRFB. The results of damage I identification with ${\mathrm{EW}}_{\mathrm{er}}$ are shown in Figure 16, and the results of damage II identification with ${\mathrm{SEW}}_{\mathrm{er}}$ are shown in Figure 17.

From Figure 16 and Figure 17, it can be seen that the damage indexes ${\mathrm{EW}}_{\mathrm{er}}$ and ${\mathrm{SEW}}_{\mathrm{er}}$ based on *D_er_* both show large mutations at the damage position, and the index values at the damage position are all greater than the damage warning values. However, the index values at non-damaged bridge pier boundary (the solidification of the pier bottom) and the pier-beam connection are also greater than the damage warning values, which does not conform to the actual situation, so the damage identification effect of ${\mathrm{EW}}_{\mathrm{er}}$ and ${\mathrm{SEW}}_{\mathrm{er}}$ is not good. In summary, compared with *D_er_*, the index based on WPNE has a stronger ability of damage identification and higher damage positioning accuracy.

### 6.2. Noise Resistance Analysis

**Figure 18:**
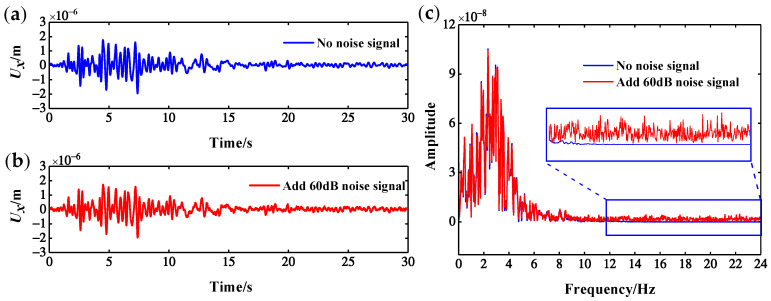
Time–frequency diagram before and after adding 60 dB noise: (**a**) no noise signal; (**b**) additional 60 dB noise signal; (**c**) frequency spectrum.

With the practical application of the damage identification method based on measured response, it can be found that the measured dynamic response signal inevitably introduces noise due to environmental (temperature, humidity) changes or equipment limitations, and the presence of noise will affect the accuracy of the damage identification method. Therefore, the damage identification method based on measured response requires a certain degree of noise immunity. To test the noise resistance of the WPNE-based identification method under noisy (or even highly noisy) conditions, the *U_x_* response measured in a noise-free environment is superimposed with zero white Gaussian noise. The *U_x_* response containing the noise is used for CCRFB damage identification. The noise level is measured by the physical signal-to-noise ratio (SNR), defined as follows [50]:

(19) $$\mathrm{SNR}=10\mathrm{lg}\frac{\sum x^{2}\left(t\right)}{\sum y^{2}\left(t\right)}$$

where x(t) is the noiseless signal, y(t) is the noise signal, SNR is the ratio of signal strength to noise intensity, and dB is a unit. SNR and noise level are inversely proportional, that is, the smaller the SNR is, the greater the noise level will be. The *U_x_* response of the CCRFB measured at the No. 63 measurement point under scenario 2 is taken as an example. Figure 18 shows the time domain and frequency spectrum before and after superimposing 60 dB noise onto the *U_x_* response of the measurement point. The figure shows that there is no obvious difference in the *U_x_* response before and after adding noise in the time domain, but there are significant differences in the frequency domain, especially in the high-frequency band (12–24 Hz). Because white Gaussian noise is a random noise, one test cannot fully evaluate the noise resistance of the damage index. Therefore, 10 separate tests were carried out under the same noise intensity, and the average of 10 test results was taken as the damage identification result under each noise level. Owing to the fact that there is a numerical difference between *EW_WPNE_* and *SEW_WPNE_* of an order of magnitude, the noise resistance of *EW_WPNE_* and *SEW_WPNE_* will be discussed separately below.

**Figure 19:**
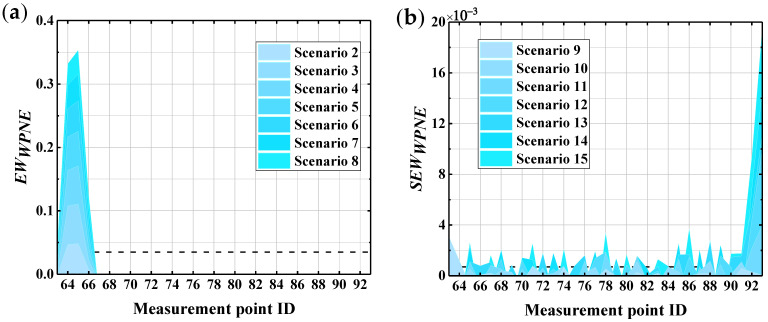
The identification results of the damage indexes when *SNR* = 60 dB: (**a**) *EW_WPNE_*; (**b**) *SEW_WPNE_*.

Figure 19 shows the results of *EW_WPNE_* and *SEW_WPNE_* damage identification in an environment with 60 dB of noise. It can be seen from the figure that *EW_WPNE_* is able to accurately identify the lower damage location in a noisy environment where SNR=60dB, with a strong noise robustness; however, for the upper damage identification, *SEW_WPNE_* suffers from many false reports and missing reports. This means that *SEW_WPNE_* with the interference of 60 dB of noise is unable to precisely locate the damage, offering poor noise resistance. The reason for this is that the construction of *SEW_WPNE_* is combined with the central difference operator (Formula (16)), indirectly introducing computational noise, thereby reducing the noise immunity *SEW_WPNE_*.

**Figure 20:**
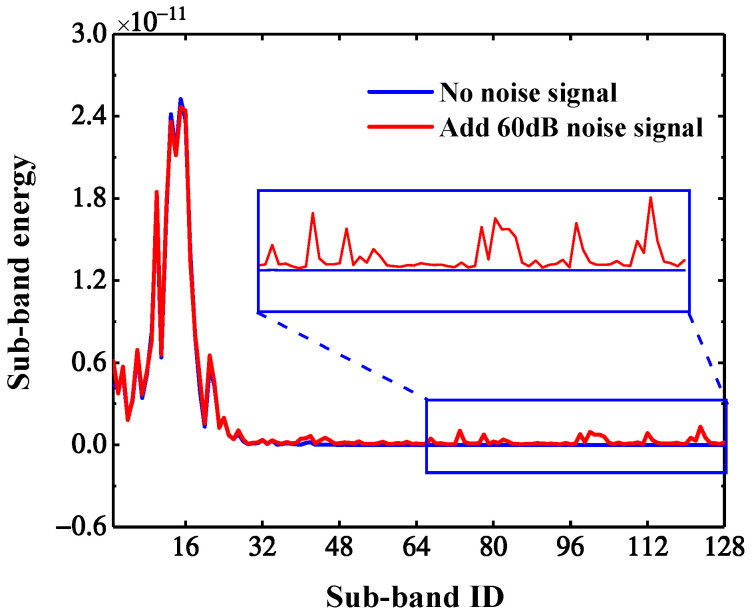
Sub-band energy ratio before and after adding 60 dB noise.

Figure 20 shows the wavelet packet energy of each sub-band of the *U_x_* response (the No. 63 measurement point under scenario 2) before and after adding 60 dB noise. According to Figure 18c and Figure 20, the noise signal, which generally has low energy and high frequency, mainly affects the high-frequency part of the response signal. After WPT, the effective information of the response is mainly in the low-frequency band, and noise information is mainly distributed in the high-frequency band, that is, the noise has a larger impact on wavelet coefficients of the high-frequency band. Therefore, selecting the top *n* low-frequency bands with high energy for the calculation of WPNE can avoid noise interference to a certain extent. According to Formulas (9) and (10), WPNE of the top *n* low-frequency bands can be expressed as:

(20) $$\mathrm{WPNE}=\overset{n}{\underset{i=1}{\sum }}-\lambda_{i}\log_{2}\left(\lambda_{i}\right)$$

where

(21) $$\lambda_{i}=\frac{L_{p}^{i}}{\sum_{i=1}^{n}L_{p}^{i}}$$

**Figure 21:**
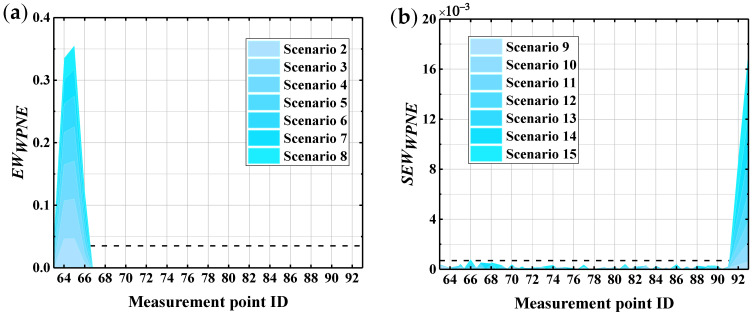
Results of the damage indexes when *SNR* = 60 and *n* = 60: (**a**) *EW_WPNE_*; (**b**) *SEW_WPNE_*.

The selection of *n* will affect the accuracy of damage identification. If *n* is too large, noise information will not be effectively removed, and if *n* is too small, individual damage information will be lost. To select an appropriate *n* value, scale correlation technology is introduced. According to the randomness of the noise and the irrelevance of the frequency band coefficients, the effective information (including individual damage information) band and the noise band are screened by analyzing the correlation of wavelet coefficients in each sub-band, that is, the effective information is mainly concentrated in frequency bands with large correlation coefficients, while noise information is concentrated in frequency bands with small correlation coefficients, and the correlation coefficient *R* between band No. *i* and band No. (i+l) is defined as follows [51]:

(22) $$R=\frac{\sum_{m=1}^{N}\left(c_{j,m}^{i}-\bar{c_{j}^{i}\left(t\right)}\right)\left(c_{j,m}^{i+l}-\bar{c_{j}^{i+l}\left(t\right)}\right)}{\sqrt{\sum_{m=1}^{N}{\left(c_{j,m}^{i}-\bar{c_{j}^{i}\left(t\right)}\right)}^{2}}\sqrt{\sum_{m=1}^{N}{\left(c_{j,m}^{i+l}-\bar{c_{j}^{i+l}\left(t\right)}\right)}^{2}}}$$

where *N* is the number of wavelet coefficients in the sub-band. In this paper, N=36, $c_{j,m}^{i}$ is the wavelet coefficient No. *m* in band No. *i* under the decomposition scale of *j*; $\bar{c_{j}^{i}\left(t\right)}$ and $\bar{c_{j}^{i+l}\left(t\right)}$ are averages of $c_{j,m}^{i}$ and $c_{j,m}^{i+l}$. The greater the correlation coefficient |R|, the greater the possibility of individual damage information being carried by a series of frequency bands. By synthesizing Figure 20 and the size of the correlation coefficient, in this paper, the top n=64 bands are chosen to construct WPNE, and ${\mathrm{EW}}_{\mathrm{WPNE}}$ and ${\mathrm{SEW}}_{\mathrm{WPNE}}$ are recalculated. In environments with 60 dB of noise, the CCRFB damage identification results based on n=64 are shown in Figure 21, where the noise resistance of ${\mathrm{SEW}}_{\mathrm{WPNE}}$ is significantly improved, so the upper damage position in noisy environments can be accurately identified and the number of false reports can be greatly reduced. In this way, automatic noise cancellation can be achieved without additional noise reduction algorithms. 

To perform quantitative analysis of the noise resistance of ${\mathrm{EW}}_{\mathrm{WPNE}}$ and ${\mathrm{SEW}}_{\mathrm{WPNE}}$ at different SNR levels, according to the Monte Carlo simulation theory, the noise resistance performance quantification index NII (Noise Immunity Index) was defined. NII is composed of Missing Report Rate (MRR) and False Report Rate (FRR), and NII is defined as follows [52]:

(23) $$\mathrm{MRR}=\frac{\sum_{i=1}^{N}a_{i}}{N}\times 100\%$$

(24) $$\mathrm{FRR}=\frac{\sum_{i=1}^{N}(1-\frac{b_{i}}{c_{i}})}{N}\times 100\%$$

(25) NII=(1−MRR)×(1−FRR)×100%

where *N* is the total number of tests, $a_{i}$ is the number of missing reports in each test, $b_{i}$ is the number of times damage is indicated correctly in each test, $c_{i}$ is the number of times the damage warning value is exceeded in each test, and i=1,2,3,…,N. NII essentially refers to the possibility of the damage index identifying the damage in the case of no missed or false reports. The greater the value of NII, the higher the accuracy and the better the noise resistance the ${\mathrm{EW}}_{\mathrm{WPNE}}$ and ${\mathrm{SEW}}_{\mathrm{WPNE}}$ will have when positioning the damage location in noisy environments. 

According to the analysis above, there is a big difference between ${\mathrm{EW}}_{\mathrm{WPNE}}$ and ${\mathrm{SEW}}_{\mathrm{WPNE}}$ with respect to noise resistance, so ${\mathrm{EW}}_{\mathrm{WPNE}}$ and ${\mathrm{SEW}}_{\mathrm{WPNE}}$ were each tested with respect to their noise robustness. ${\mathrm{EW}}_{\mathrm{WPNE}}$ was tested 500 times in environments where SNR={50dB, 40dB, 30dB, 20dB}, and the results of ${\mathrm{EW}}_{\mathrm{WPNE}}$ noise resistance analysis are presented in Table 3. Similarly, ${\mathrm{SEW}}_{\mathrm{WPNE}}$ was tested 500 times with SNR={70dB,60dB,50dB,40dB}, and the results of the noise resistance analysis are presented in Table 4. The following conclusion can be drawn on the basis of an observation of the two tables: (1) MRR of the ${\mathrm{EW}}_{\mathrm{WPNE}}$ index is generally less than 5%, so the damage position can be easily identified. In terms of damage identification accuracy, in environments with 30dB of noise, each NII of ${\mathrm{EW}}_{\mathrm{WPNE}}$ is greater than 90%, indicating that ${\mathrm{EW}}_{\mathrm{WPNE}}$ is suitable for identifying lower damage of the CCRFB in noisy environments where SNR ≥ 30dB; (2) ${\mathrm{SEW}}_{\mathrm{WPNE}}$ is much worse than ${\mathrm{EW}}_{\mathrm{WPNE}}$ in terms of noise resistance. Both MRR and FRR under the scenario of slight damage are greater than those of ${\mathrm{EW}}_{\mathrm{WPNE}}$. In environments with 50 dB of noise, each NII of ${\mathrm{SEW}}_{\mathrm{WPNE}}$ is larger than 90%, so it is suitable for identifying upper damage to the CCRFB in noisy environments where SNR ≥ 50dB. In summary, the damage identification method based on WPNE has high noise robustness, and other filtering algorithms are not required. By selecting the top n=64 bands to calculate WPNE, independent noise reduction can be achieved, and the location of structural damage in noisy environments where SNR ≥ 50dB can be identified.

### 6.3. Effect of Seismic Excitation

To illustrate that the proposed method is not affected by the type of seismic excitation, the measured dynamic response is normalized in the following way [52]:

(26) $${\mathrm{ITF}}_{m/n}=\mathrm{iFT}\left(\frac{{\mathrm{FT}(U}_{x}^{m})}{{\mathrm{FT}(U}_{x}^{n})}\right)$$

where $U_{x}^{m}$ and $U_{x}^{n}$ represent the displacement response of the *m*-th and the *n*-th measurement points in the *x* direction, respectively. FT stands for Fourier Transform and iFT stands for inverse Fourier Transform. The essence of ${\mathrm{FT}(U}_{x}^{m})/{\mathrm{FT}(U}_{x}^{n})$ is the transmissibility function (TF), and the essence of ${\mathrm{ITF}}_{m/n}$ is the inverse transmissibility function (ITF); ${\mathrm{ITF}}_{m/n}$ (a temporal signal) obtained using Equation (26) eliminates the effect of the excitation force.

**Figure 22:**
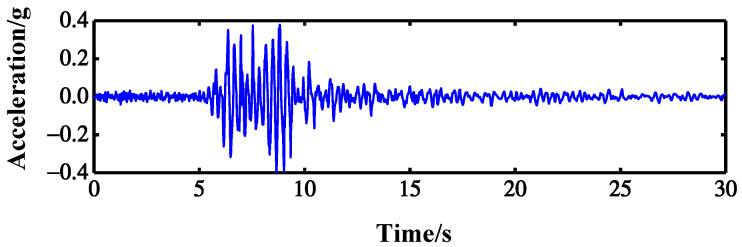
Accelerogram of WN earthquake.

**Figure 23:**
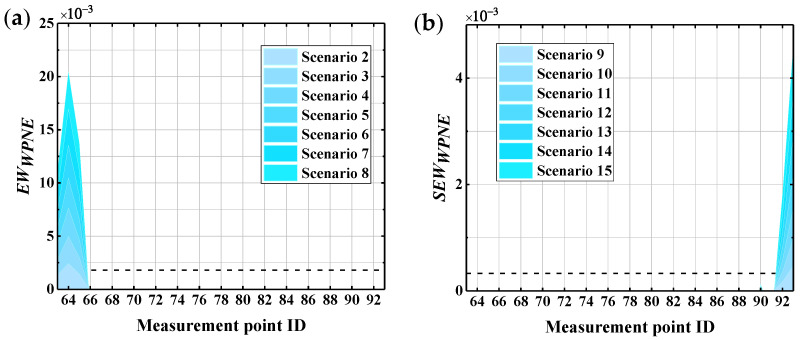
Damage identification results of the CCRFB under WN seismic excitation: (**a**) *EW_WPNE_*; (**b**) *SEW_WPNE_*.

Uniformly selecting $U_{x}^{78}$ as the reference response, damage identification of CCRFB under WN (Whittier Narrows) seismic excitation was carried out using ${\mathrm{ITF}}_{63/78}$, ${\mathrm{ITF}}_{64/78}$,…, ${\mathrm{ITF}}_{93/78}$ after eliminating the effect of excitation force. The accelerogram of WN is shown in Figure 22 and Figure 23 presents the identification results of damage I and II. It can be clearly seen from the figure that ${\mathrm{EW}}_{\mathrm{WPNE}}$ and ${\mathrm{SEW}}_{\mathrm{WPNE}}$ can still accurately identify the damage position, indicating that the method based on WPNE is not affected by seismic excitation type. The proposed method is universally applicable for different types of seismic excitation.

## 7. Conclusions

In this paper, the seismic damage identification indexes ${\mathrm{EW}}_{\mathrm{WPNE}}$ and ${\mathrm{SEW}}_{\mathrm{WPNE}}$ are constructed based on WPNE and applied in the study of seismic damage identification of CCRFBs under seismic excitation. The numerical simulation results show that ${\mathrm{EW}}_{\mathrm{WPNE}}$ and ${\mathrm{SEW}}_{\mathrm{WPNE}}$ are able to accurately identify the location of the damage and have good monotonicity with damage severity. Moreover, the advantages of the WPNE-based method are further illustrated through comparison with the $D_{\mathrm{er}}$-based method. The noise robustness analysis shows that when the first 64 bands are selected to calculate the WPNE, the damage index does not require other noise reduction algorithms to identify structural damage in noisy environments with SNR ≥ 50dB. In addition, we find that the proposed method is not affected by the type of seismic excitation and that the damage index still accurately indicates the location of damage to CCRFBs even when structural damage identification is performed using responses with the effects of the excitation forces removed. Therefore, when combined with advanced sensing techniques, the WPNE-based method holds significant promise in civil engineering for damage detection in special-shaped bridges.

## Figures and Tables

**Table 1:** Damage scenarios of the CCRFB.

Damage Location	Stiffness Reduction Rate	Damage Scenarios	Damage Location	Stiffness Reduction Rate	Damage Scenarios
I, II	0%	1			
I	5%	2	II	5%	9
I	10%	3	II	10%	10
I	15%	4	II	15%	11
I	20%	5	II	20%	12
I	25%	6	II	25%	13
I	30%	7	II	30%	14
I	35%	8	II	35%	15

**Table 2:** Damage warning values for the CCRFB.

	Lower Damage I (*EW_WPNE_*)	Upper Damage II (*SEW_WPNE_*)
Damage warning value	3.8000 × 10^−2^	5.9645 × 10^−4^

**Table 3:** *EW_WPNE_* noise resistance analysis results.

Scenario	*MRR*(%)				*FRR*(%)				*NII*(%)			
	*SNR*(dB)				*SNR*(dB)				*SNR*(dB)			
	20	30	40	50	20	30	40	50	20	30	40	50
2	8.92	2.03	0.00	0.00	14.42	7.76	2.56	0.00	77.95	90.37	97.44	100.00
3	5.21	1.70	0.00	0.00	10.27	5.47	0.80	0.00	85.06	92.92	99.20	100.00
4	1.09	0.04	0.00	0.00	8.71	3.28	0.10	0.00	90.29	96.68	99.90	100.00
5	0.02	0.00	0.00	0.00	3.55	1.29	0.00	0.00	96.43	98.71	100.00	100.00
6	0.00	0.00	0.00	0.00	1.08	1.06	0.00	0.00	98.92	98.94	100.00	100.00
7	0.00	0.00	0.00	0.00	0.53	0.25	0.00	0.00	99.47	99.75	100.00	100.00
8	0.00	0.00	0.00	0.00	0.09	0.00	0.00	0.00	99.91	100.00	100.00	100.00

**Table 4:** *SEW_WPNE_* noise resistance analysis results.

Scenario	*MRR*(%)				*FRR*(%)				*NII*(%)			
	*SNR*(dB)				*SNR*(dB)				*SNR*(dB)			
	40	50	60	70	40	50	60	70	40	50	60	70
9	12.34	4.55	1.07	0.48	17.29	3.34	2.45	1.12	72.50	92.26	96.51	98.40
10	9.28	2.29	0.02	0.00	13.70	1.46	0.43	0.20	78.29	96.28	99.55	99.80
11	7.90	1.15	0.00	0.00	10.55	0.83	0.02	0.00	82.38	98.03	99.98	100.00
12	3.89	0.05	0.00	0.00	7.01	0.46	0.00	0.00	89.37	99.49	100.00	100.00
13	0.77	0.00	0.00	0.00	4.96	0.01	0.00	0.00	94.31	99.99	100.00	100.00
14	0.06	0.00	0.00	0.00	3.01	0.00	0.00	0.00	96.93	100.00	100.00	100.00
15	0.00	0.00	0.00	0.00	2.11	0.00	0.00	0.00	97.89	100.00	100.00	100.00

## Data Availability

Not applicable.
